# A psychological insight of Moroccan adults’ immunisation behaviour towards emergency vaccines

**DOI:** 10.4102/phcfm.v16i1.4353

**Published:** 2024-05-03

**Authors:** Nour El Houda Benkaddour, Hind Khalil, Asmae Lekfif, Naima Abda, Bouchra Oneib, Yassamine Bentata

**Affiliations:** 1 Laboratory of Epidemiology, Clinical Research and Public Health, Faculty of Medicine and Pharmacy, Mohammed First University, Oujda, Morocco; 2 Maternal-Infant and Mental Health Research Laboratory, Faculty of Medicine and Pharmacy, Mohammed First University, Oujda, Morocco; 3 Department of Psychiatry, Mohammed VI University Hospital, Oujda, Morocco; 4 Nephrology and Kidney Transplantation Unit, Mohammed VI University Hospital, Oujda, Morocco

**Keywords:** vaccination behaviour, stress, COVID-19, young adults, university students, Morocco

## Abstract

**Background:**

The psychology of vaccination behaviour explains how thoughts and feelings influence people’s willingness to receive vaccines. Understanding vaccination behaviour is crucial to successfully managing vaccination campaigns.

**Aim:**

Investigating factors associated with immunisation stress among students at Mohammed First University.

**Setting:**

This study was conducted on students at Mohammed First University institutions.

**Methods:**

This study is a descriptive and analytical cross-sectional study. It was conducted on 305 students at Mohammed First University institutions using a 90-item questionnaire.

**Results:**

Three hundred and five participants have been included in this survey. Overall, 65.5% of the students in our sample had a positive perception towards COVID-19 vaccines. Nevertheless, 34.5% had a negative opinion regarding immunisation. According to the analysis of perceived stress scale, 40% (*n* = 122) of students expressed moderate to high stress regarding vaccination. Students with a negative perception of vaccine showed a higher level of stress than those with a positive one. Stressed students tended to be older than others, coming from other institutions, other than the medical faculty, and were renting alone. Vaccine accessibility was the less significant reason associated with stress regarding vaccination. Moreover, participants with high levels of confidence in social media, exhibited higher stress. Nevertheless, those who believed in scientific journals were significantly less stressed.

**Conclusion:**

These results reflect a positive perception and acceptance of vaccines, with a considerable level of stress regarding vaccination.

**Contribution:**

This study suggests emphasising the mental health of Moroccan young adults, to better sensitise and inform them about immunisation.

## Introduction

The man–pandemic relationship has, since antiquity, marked humanity’s history, notably through their conflicting nature. The development of our current healthcare system was prompted by epidemics and pandemics, which wiped out entire populations in a matter of months or even days. The birth of immunisation truly revolutionised the management of infectious diseases. Yet, its principle of preventing infection by infection has been a source of apprehension, reluctance and polemics among the general population since Pasteur’s first essays.

Having received emergency approval for use of a few severe acute respiratory syndrome coronavirus 2 (SARS-CoV-2) vaccines worldwide, the vaccines have been greeted with either an active request, passive acceptance or a definite refusal. Indeed, given the urgent nature of the pandemic, the rapid development of COVID-19 vaccines has contributed to numerous concerns, such as those related to short-term side effects (thromboembolic events, sudden death, etc.).^1^ Eventual long-term side effects are not well-understood yet, and are a source of apprehension and public controversy.

COVID-19 vaccine uptake rates are widely variable, from a positive rate of 83.6% in Saudi Arabia and 87% in Lebanon, to an extremely negative one of 34.9% in Egypt and 15.4% in Cameroon.^2,3,4,5,6^ This could be attributable to several factors including the level of pandemic severity in the concerned country and the historical, as well as political and socio-cultural context.^7,8^ Accordingly, research into each country’s perception of immunisation is paramount to determine influences on it, and to attempt an appropriate response.

Since the start of vaccination campaigns, Morocco has bet on young adults’ vaccination to achieve generalised immunity, as the Moroccan age profile is rather young. Besides, these young people often encounter difficulties in respecting permanent protection measures, and thus, they are considered as a major source of transmission of the virus, especially to their elderly family members. Advanced understanding of young adults’ perspective towards vaccination, and potential factors influencing their vaccine intention, will contribute to ongoing development and implementation of effective strategies, to promote SARS-CoV-2 vaccine adherence among this group. Yet, and according to our literature search, limited data are available in Morocco on the perceived value of vaccination against COVID-19. Therefore, it is important to investigate this issue to address vaccine hesitancy, the major challenge for vaccination campaigns.

Furthermore, exploring whether the SARS-CoV-2 vaccine is accepted as an emergency vaccine is crucial. Indeed, such a survey is exemplary to better approach, sensitise and inform the population for vaccination in case of an eventual new pandemic, especially in the current situation of the climate change crisis; a perfect niche for viruses susceptible to be transmitted to humans.^9,10^

In the realm of vaccine behaviour, the psychological science can provide new insights, providing systems and policy development support to directly facilitate health system action.^11^ The psychology of vaccination behaviour explains how thoughts and feelings influence people’s willingness to receive vaccines. Posterior studies indicate that perceived risk of infectious disease, perceived efficacy, and safety of the vaccine are correlated with vaccine acceptance.^11^

At the same time, vaccination programmes could be suspended because of immunisation stress reactions. Indeed, people are considered to be more aware when new vaccines are first proposed, and this may lead to hesitancy about vaccines, and a loss of public confidence may result when such mass events occur.^12,13,14^

Hence, the aim of the present study was to investigate vaccine perception and factors associated with immunisation stress among Mohammed First University’s students.

## Research methods and design

### Study design

We opted for a descriptive and analytical cross-sectional study, conducted during the period from February 2022 to October 2022. The survey was conducted on students enrolled at Mohammed First University, a group of 10 institutions based mainly in the Eastern region of Morocco in Oujda, via face-to-face surveys, using a 90-item questionnaire in the French language.

### Sample size

The selected sample was calculated online through a free and open software program called: ‘*Open Source Statistiques Épidémiologiques pour la Santé Publique*’.^15^ For an error risk: α = 5%, an expected proportion of positive perception: *p* = 82%,^16^ and a population size: *N* = 77 886 students, the minimum number of students to be included is 227.

A non-probability sampling procedure called snowball was used. In fact, the data were collected through students and teachers contacts, by circulating the survey questionnaire to people matching the required characteristics. They were then asked to suggest other people with a similar profile.

### Questionnaire

We designed a questionnaire, composed of 10 parts. The first section is a description of the survey and its objectives. The questionnaire itself begins in the second part. It includes demographic data (gender, age, city of residence, education level, etc.), information related to SARS-CoV2 vaccination, including vaccination profile and perception of vaccines recommended by public health authorities. We have also collected information regarding COVID-19 disease. The remaining part of the questionnaire focuses on the measurement of some vaccine anxiety disorders (anxiety, perceived stress and post-traumatic stress disorders [PTSD]) and insomnia, by using four questionnaires mentioned further in the text:

- First scale: The severity of anxious symptoms was scored according to the Hamilton scale.^17^ This ladder measures the seriousness of anxiety symptoms on 14 parameters such as anxious humour, fears, insomnia, depressed humour, etc.
- Second scale: Perceived stress level was evaluated using the Perceived Stress Scale (PSS).^18^ The 10-item PSS questionnaire provides a very popular and widely used tool to understand how different situations affect feelings and perceived stress.
- Third scale: PTSD was assessed using the post-traumatic stress disorder Checklist Scale (PCLS).^19^ This scale is simple and easy to complete. It is very useful for screening for PTSD in clinical and research settings. The scale consists of 17 items, rating the level of each component of the 17 associated symptoms.
- Fourth scale: The Insomnia Severity Index (ISI) scale was used to evaluate insomnia.^20^ This short seven-item scale, allows the assessment of insomnia by determining satisfaction of sleep, daily performance, and thus anxiety regarding sleep difficulties.

Volunteers were asked to test the questionnaire in order to estimate how long it would take them to complete it, and thus to modify any incomprehensible question. Our pre-test evaluation of the questionnaire, allowed us to estimate a duration of approximately 15 min for each student. Therefore, we decided to conduct the interviews during their break.

### Statistical analysis

The quantitative variables were presented as a mean and its standard deviation, whereas the categorical ones were described as a percentage.

The unpaired samples *T* test was applied to compare quantitative variables. The Chi-square (χ^2^) test or Fisher’s exact test were used to compare qualitative variables. A *p*-value inferior than 0.05 was deemed significant for this survey. Variables exhibiting a significance threshold of *p* ≤ 0.05 were included in the backward stepwise logistic regression model. The IBM SPSS Statistics software, Version 21.0, supported all statistical analyses.

### Ethical procedures

The protocol of this study was examined and approved by the Ethics Review Committee for Biomedical Research of the Faculty of Medicine and Pharmacy of Oujda (CERBO) according to the guidelines the Helsinki Declaration prepared. The submission file is registered and approved under the order number: 36/2021.

A written consent was obtained for each participant prior to the beginning of the investigation. All participants were informed about the objectives of the study, its voluntary and anonymous nature, and the confidentiality of its information.

## Results

### Background and demographic profile of the sample

Three hundred and five copies were collected from the 350 that were distributed, yielding a response rate of 87.1%.

The female population represented 51.3% of the total sample, with a female to male ratio of 1.05. The mean age was 22.62 ± 5.07 years. Among the 305 students, more than 90% were urban dwellers, 47.1% of whom were from Oujda, the capital of the Eastern Region of Morocco.

According to their vaccination profile, 87.2% of participants received their first and second doses of the vaccine, and only 14.7% were vaccinated with the third dose. The remaining 12.8% of respondents did not receive any vaccine and were therefore considered unvaccinated.

Over 65% (*n* = 199) of the students in our sample had a positive perception towards the first two doses of the vaccine. Nevertheless, 73% had a negative one regarding the third dose, whereas a better perception of the vaccine doses was reported amongst vaccinated respondents (71.7% of vaccinated students had a positive perception of the first two doses).

Concern over potential side effects was identified as the most common reason for refusal or hesitation to undergo vaccination (5.2%), while obligation to do so, as well as self and others protection, were the most common acceptance motives.

In terms of students’ opinions on vaccines recommended by public health authorities, 43% believe such vaccines are useful, 37.7% assume their effectiveness, and 35.4% indicate that their safety could be presumed. Yet, 51.1% of all participants clearly indicated that there was no public acceptance of the COVID-19 vaccines. Besides, 39% of students expressed their confidence in doctors and 43.8% in scientific journals as a source of information regarding immunisation. Furthermore, television news (50.5%), social networks (56.1%), family, friends and acquaintances (51.3%) were not deemed as a reliable source.

Tables 1, Table 2 and Table 3 summarise the descriptive results of data distribution in the sample.

**TABLE 1:** Sociodemographic characteristics of the respondents (*N* = 305).

Variables	*n*	%
**Gender**		
Female	154	51.3
Male	146	48.7
**Region of residence**		
Urban	297	98.7
Rural	4	1.3
**Establishment**		
Faculty of Sciences	111	36.4
Faculty of Medicine	66	21.6
Others	128	42.0
**Study level**		
Junior grade	147	48.2
Middle grade	130	42.6
Senior grade	28	9.2
**Accommodation**		
Family	173	57.9
University campus	22	7.4
Alone in a rental	54	18.1
With other renters	50	16.7
**Chronic diseases**		
No	229	75.1
Yes	76	24.9
**Previous COVID-19**		
Yes	123	40.3
No	182	59.7
**SARS CoV-2 infection awareness**		
Yes, a lot	18	5.9
Yes, quite	139	45.6
Yes, a little	131	43.0
No, I have no information	17	5.6
**Compliance with sanitary measures**		
Always	34	11.1
Often	50	16.4
Sometimes	98	32.1
Rarely	55	18.0
Never	68	22.3
**Source of COVID-19 information**		
Establishment	78	25.6
Radio	42	13.8
Television	169	55.6
Social networks	254	83.6
Others	27	8.9

SARS-CoV-2, severe acute respiratory syndrome coronavirus 2.

Note: Age mean ± standard deviation= 22.62 ± 5.07.

**TABLE 2:** Vaccination profile and perception of COVID-19 vaccination (*N* = 305).

Variables	*n*	%
**Vaccination profile (1st and 2nd doses)**		
Yes	266	87.2
No	39	12.8
**Vaccination profile (3rd dose)**		
Yes	39	14.7
No	195	73.3
Planned for the next few days	32	12.0
**Vaccine type (1st and 2nd doses)**		
Non-replicating viral vector	51	19.2
RNAm	10	3.8
Inactivated virus	207	77.8
**Vaccine type (3rd dose)**		
Non-replicating viral vector	5	12.8
RNAm	11	28.2
Inactivated virus	23	59.0
**Side effects**		
Mild to moderate	150	56.4
Severe	0	0.0
No side effects	116	43.6
**Refusal or hesitation reasons**		
Vaccine is not very effective	13	4.3
Fear of eventual side effects	16	5.2
For no good reason	13	4.3
Others	6	2.0
**Acceptance reasons**		
Self-protection	148	48.5
Protecting others	137	44.9
Recommendation from the health care community and/or national guidelines	64	21.0
Recommendation from family and/or friends and/or colleagues	39	12.8
Vaccine accessibility	49	16.1
Obligation to do so	152	49.8
Others	2	0.7
**Vaccination perception (1st and 2nd dose)**		
Positive perception	199	65.5
Negative perception	105	34.5
**Vaccination perception (3rd dose)**		
Positive perception	82	27.0
Negative perception	222	73.0

**TABLE 3:** Vaccine opinions and participants’ confidence in informational sources regarding vaccination (*N* = 305).

Variables	*n*	%
**Vaccines recommended by public health authorities are useful**		
I agree	131	43.0
I disagree	63	20.6
I do not know	111	36.4
**Vaccines recommended by public health authorities are effective**		
I agree	115	37.7
I disagree	84	27.5
I do not know	106	34.8
**Vaccines recommended by public health authorities are safe**		
I agree	108	35.4
I disagree	90	29.5
I do not know	107	35.1
**Vaccines recommended by the public health authorities have been accepted by the population**		
I agree	78	25.6
I disagree	156	51.1
I do not know	71	23.3
**Doctors**		
Very confident	119	39.0
Moderately confident	141	46.2
Not at all confident	45	14.8
**Official websites**		
Very confident	54	17.7
Moderately confident	161	52.8
Not at all confident	90	29.5
**Television news**		
Very confident	24	7.9
Moderately confident	127	41.6
Not at all confident	154	50.5
**Social networks**		
Very confident	19	6.2
Moderately confident	115	37.7
Not at all confident	171	56.1
**Scientific journals**		
Very confident	133	43.8
Moderately confident	126	41.4
Not at all confident	45	14.8
**Family, friends, acquaintances**		
Very confident	36	11.8
Moderately confident	112	36.8
Not at all confident	156	51.3

The severity of insomnia symptoms and anxiety regarding vaccination were also investigated. According to the scales analysis, 11.1% (*n* = 34) of students had moderate to severe anxiety, 40% (*n* = 122) expressed moderate to high level of stress, and 4.9% (*n* = 15) had a moderate level of insomnia towards vaccination. The rates of some post-traumatic stress disorder symptoms are detailed in Table 4.

**TABLE 4:** Anxiety, perceived stress, post-traumatic stress disorder, and insomnia levels regarding vaccination (*N* = 305).

Variables	*n*	%
**Anxiety level**		
Normal anxiety	225	73.8
Mild anxiety	46	15.1
Moderate anxiety	16	5.2
High to severe anxiety	18	5.9
**Perceived stress level**		
Low perceived stress	183	60.0
Moderate perceived stress	110	36.1
High perceived stress	12	3.9
**Level of some PTSD symptoms**		
Repeated, disturbing memories, thoughts, or images of a stressful experience from the past	73	23.9
Repeated, disturbing dreams of a stressful experience from the past	17	5.6
Feeling very upset when something reminded you of a stressful experience from the past	31	10.2
Having physical reactions (e.g. heart pounding, trouble breathing, or sweating) when something reminded you of a stressful experience from the past	11	3.6
Avoid thinking about or talking about a stressful experience from the past or avoid having feelings related to it	46	15.1
Avoid activities or situations because they remind you of a stressful experience from the past	64	21.0
Loss of interest in things that you used to enjoy	29	9.5
Trouble falling or staying asleep	12	3.9
Feeling irritable or having angry outbursts	13	4.3
Having difficulty concentrating	11	3.6
Feeling jumpy or easily startled	18	5.9
**Insomnia level**		
**No insomnia**	240	78.7
**Sub-clinical insomnia (mild)**	50	16.4
**Clinical insomnia (moderate)**	15	4.9
**Clinical insomnia (severe)**	-	-

PTSD, Post-Traumatic Stress Disorder.

### Participants’ characteristics according to stress level related to vaccination

The impact of sample characteristics on the level of stress regarding vaccination is reported in Table 5.

**TABLE 5:** Univariate and multivariate analysis of factors influencing stress regarding COVID-19 vaccination (*N* = 305).

Variables	Present stress		*p*-value	OR	95% CI	*p*-value
	*n*	%				
**Gender**						
Female	57	37.0	0.33	-	-	-
Male	62	42.5	-	-	-	-
Age*	-	-	0.003	1.09	1.03–1.15	0.002
**Region of residence**						
Urban	118	39.7	1	-	-	-
Rural	1	2.05	-	-	-	-
**Establishment**						
Faculty of Sciences	48	43.2	0.01	-	-	-
Faculty of Medicine	16	24.2	-	-	-	-
Others	58	45.3	-	-	-	-
**Study level**						
Junior grade	55	37.4	0.32	-	-	-
Middle grade	58	44.6	-	-	-	-
Senior grade	9	32.1	-	-	-	-
**Accommodation**						
Family	58	33.5	0.001	1	-	-
With other renters	17	34.0	-	1.20	0.59–2.42	0.60
University campus	12	54.5	-	2.49	0.97–6.36	0.05
Alone in a rental	33	61.1	-	3.74	1.87–7.44	< 0.0001
**Chronic diseases**						
No	87	38.0	0.21	-	-	-
Yes	35	46.1	-	-	-	-
**Previous COVID-19**						
Yes	45	36.6	0.34	-	-	-
No	77	42.3	-	-	-	-
**SARS CoV-2 infection awareness**						
Yes, a lot	6	33.3	0.34	-	-	-
Yes, quite	52	37.4	-	-	-	-
Yes, a little	54	41.2	-	-	-	-
No, I have no information	10	58.8	-	-	-	-
**Vaccination profile (1st and 2nd doses)**						
Yes	109	41.0	0.36	-	-	-
No	13	33.3	-	-	-	-
**Vaccination profile (3rd dose)**						
Yes	18	46.2	0.02	-	-	-
No	85	43.6	-	-	-	-
Planned for the next few days	6	18.8	-	-	-	-
**Side effects**						
Mild to moderate	66	44.0	0.25	-	-	-
Severe	0	0.0	-	-	-	-
No side effects	43	37.1	-	-	-	-
**Refusal or hesitation reasons**				-	-	-
Vaccine is not very effective	6	46.2	0.77	-	-	-
Fear of eventual side effects	6	37.5	1	-	-	-
For no good reason	2	15.4	0.08	-	-	-
Others	4	66.7	0.22	-	-	-
**Acceptance reasons**						
Self-protection	55	37.2	0.35	-	-	-
Protecting others	45	32.8	0.02	-	-	-
Recommendation from the health care community/national guidelines	19	29.7	0.06	-	-	-
Recommendation from family, friends, colleagues	16	41.0	1	-	-	-
Vaccine accessibility*	9	18.4	0.001	3.65	1.62–8.21	0.002
Obligation to do so	61	40.1	1	-	-	-
Others	0	0.0	0.51	-	-	-
**Vaccination perception**						
Positive perception	73	36.7	0.09	-	-	-
Negative perception	49	46.7	-	-	-	-
**Doctors**						
Very confident	46	38.7	0.79	-	-	-
Moderately confident	56	39.7	-	-	-	-
Not at all confident	20	44.4	-	-	-	-
**Official websites**						
Very confident	22	40.7	0.69	-	-	-
Moderately confident	61	37.9	-	-	-	-
Not at all confident	39	43.3	-	-	-	-
**Television news**						
Very confident	9	37.5	0.46	-	-	-
Moderately confident	56	44.1	-	-	-	-
Not at all confident	57	37.0	-	-	-	-
**Social networks**						
Very confident	10	52.6	0.01	2.82	0.98–8.12	0.05
Moderately confident	56	48.7	-	2.31	1.35–3.95	0.002
Not at all confident	56	32.7	-	1	-	-
**Scientific journals**						
Very confident	41	30.8	0.01	-	-	-
Moderately confident	60	47.6	-	-	-	-
Not at all confident	20	44.4	-	-	-	-
**Family, friends, acquaintances**						
Very confident	15	41.7	0.34	-	-	-
Moderately confident	50	44.6	-		-	-
Not at all confident	56	35.9	-	-	-	-

SARS-CoV-2, severe acute respiratory syndrome coronavirus 2.

Note: Age mean ± standard deviation = 23.78 ± 6.41.

* In the multivariate analysis, we took the answer Yes as a reference.

The *p* level at which the results are significant is *p* 0.05.

Although women were less exposed to stress when compared to men, yet the difference between the two groups was not significant (*p* = 0.33). The stressed students tended to be older (mean age ± standard deviation in years: 23.78 ± 6.41 vs. 21.86 ± 3.77, *p* = 0.003), from establishments other than medical faculty (*p* = 0.01), and who lived alone as tenants (*p* = 0.001).

On the other hand, and according to participants’ vaccination profile, the respondents who received their third dose of the SARS-CoV-2 vaccine presented a significantly higher level of perceived stress than those who did not receive this dose or who were interested in receiving it (*p* = 0.02).

Protecting others (*p* = 0.02) and vaccine accessibility (*p* = 0.001) were the less significant reasons associated with stress regarding vaccination. However, no significant difference was found concerning refusal or hesitation reasons about COVID-19 vaccination.

In terms of perception, students with a negative experience with the first two doses expressed a more remarkable level of stress, than those with a positive perception. Nevertheless, this difference was insufficiently significant (*p* = 0.09).

We note that participants with a high level of confidence in social networks as an informational source regarding immunisation displayed higher stress compared to those with less faith (*p* = 0.01). Meanwhile, students who believed in scientific journals were found to be less stressed (*p* = 0.01).

### Influencing factors related to COVID-19 vaccination stress

Binary logistic regression analysis revealed that age (OR 1.09, 95% CI [1.03–1.15], *p* = 0.002), being alone in a rental (OR 3.74, 95% CI [1.87–7.44], *p* < 0.0001), accepting the vaccine for any reason other than its accessibility (OR 3.65, 95% CI [1.62–8.21], *p* = 0.002), and confidence in social networks as an informational source for immunisation (OR 2.31, 95% CI [1.35–3.95], *p* = 0.002), were significantly associated with stress. The results of the binary logistic regression analysis of risk factors related to the occurrence of vaccination stress are summarised in Table 5.

## Discussion

Whereas vaccination might seem to be a simple reflex, in reality, it depends on a combination of different factors. Indeed, assessment of disease risk, vaccine confidence and motivation to get vaccinated are all associated with vaccination behaviour, and each of these components is influenced by several factors.^11^

The present study was conducted to identify and comprehend the elements influencing the psychological aspects of vaccination behaviour, which is considered to be the main factor allowing interventions on individuals’ willingness to be vaccinated. This pattern of behaviour was evident by studying the factors influencing the ‘Vaccine Confidence’ component, notably stress about vaccination.

### The impact of sociodemographic characteristics of the respondents on the level of stress regarding vaccination

Results analysis of potential stress-related factors regarding vaccination indicated that stressed responders tended to be older. A study conducted by Rogowska et al.^21^ revealed that vaccinated students were older and exhibited high levels of stress and fear related to COVID-19 compared to the unvaccinated group. We note that, in the present work, the percentage of stressed students in the vaccinated group was higher than that among the unvaccinated ones, although the difference was statistically not significant. Therefore, further research is needed to elucidate this relationship. This would suggest that older students may pay more attention to their own safety when dealing with new vaccines. It is possible that older students may have different educational or risk perception levels, and thus are more conscious during emergency situations.^22^

The relationship between gender and immunisation behaviour has been reported in several previous studies.^23,24,25,26^ Gender-related behaviours may influence variations in vaccine acceptance and perception within the adult population.^26^ A study assessing associations between mental health and intent to be vaccinated against COVID-19 revealed that women were found to be more anxious than men.^23^ Yet another survey showed more negative attitudes towards such vaccines among men.^27^ In terms of physiology, it appears that women are more likely to present side effects than men after immunisation.^28,29^ Accordingly, they report being more concerned about vaccine safety and efficiency than men, which may contribute to a lower vaccination rate among women.^30^ In our study, we found that female students developed less stress regarding SARS-CoV-2 vaccines compared with male participants. This finding may be explained by the frequent use of preventive care services by women to the detriment of men.^31^

The results highlighted a significant difference between stress level presented by medical students compared to students from other institutions (*p* = 0.01). The evidence suggests that medical students appear to be considerably less apprehensive about vaccination. These findings are coherent with previous studies.^32,33,34^ One explanation for this might be the nature of their background, where a more clinical approach is required. Therefore, medical students would have the opportunity to acquire greater insight, and have a greater sense of responsibility for themselves and for others.

We evaluated the impact of housing on the level of stress towards vaccination. As a result, our findings indicate that students who lived alone in a rental presented a significant increased level of stress compared to the others. It seems that students staying alone in a rental are experiencing a deeper sense of loneliness than those living with others. Previous investigations highlighted the association between the lack of social connection, depression and social anxiety, as well as reduced vaccine use.^35,36^ Consequently, this connection between social link and psychological well-being ought to be considered to improve the adherence of this group within the next vaccination programmes, while preserving their mental health.

### The impact of reasons for accepting vaccination on the level of stress regarding vaccination

Protecting others and vaccine accessibility were reasons for acceptance, significantly less associated with stress (*p* = 0.02, *p* = 0.001). A previous study conducted among Chinese university students revealed the reasons behind their vaccination behaviours.^2^ Most of their respondents who accepted vaccination indicated that perceived benefits of vaccination for themselves prompted them to do so, followed by their perceptions of decreased contamination risks for others. This suggests that university students are more conscious when faced with emergency situations, expressing a high level of social responsibility, and therefore positively influence the vaccine perception and acceptance.

Since the inoculation campaigns began, Morocco has been actively working to facilitate the access to vaccination services by establishing local centres and providing free vaccine to the entire population. On the other hand, and as a young population, university students benefited from the opportunity to get vaccinated in a period where many types of vaccines were already available. Thus, they were able to decide when, where and what type of vaccine they would receive. Hence, it is possible that the accessibility and availability of such vaccines might provide a double benefit in terms of university students’ vaccination behaviour, allowing them to express particular, rather positive, emotions while accepting this action.

### The impact of vaccination profile and COVID-19 vaccination perception on the level of stress regarding vaccination

A further outcome relevant to our study is the booster dose. Indeed, the percentage of stressed people in the group that received the third dose was significantly higher (*p* = 0.02). These results are at odds with another publication, whereby respondents who were willing to receive such a dose expressed better mental health.^7^ Previous studies have indicated that post-vaccination side effects, inability to tolerate the first two doses’ adverse reactions, and negative perception of the booster dose’s benefits were among the most cited reasons for refusing the third dose.^37,38,39^ Apparently, although some students accepted the booster dose for different reasons, their attitude towards this so-called dose might not necessarily be positive. Accordingly, further investigations are required to highlight and understand the relationship between the mental health status and the booster dose.

We note that side effects were somewhat more susceptible to be present within the stressed group as compared to those who exhibited low, or no, stress regarding vaccination. In fact, stress-related responses following vaccination are not considered as new insights.^13^ Indeed, vaccination refers to the process of vaccine administration, namely the pain involved in the shot, the presence of needles and or blood, the waiting line, and others’ reactions in the post-vaccination waiting room.^13,40^ All these events can cause psychological distress.^40^ In addition, these immunisation stress-related responses are more susceptible when new vaccines first appear, and may promote vaccine hesitancy.^13,14,40^ Therefore, further investigations are required to identify individuals at highest risk for developing stress-related adverse events and thus attempt to uncover potential predictors.

No significant relationship between stress and vaccination perception was observed in our study. Yet, previous studies have noted the presence of an eventual association between these two variables. Students with high stress levels, and who reported more severe depressive symptoms, were more susceptible to have a negative perception of the SARS-CoV-2 vaccines.^41^ Further studies indicated that individuals with poor mental health had decreased intentions to adopt health-related behaviours.^42,43^ It seems that stress can prompt negative cognitive responses, such as negative behavioural ones.^41^ The non-significance of our results could be attributed to the period when the survey was performed. Indeed, prior studies have demonstrated that vaccine intent changes over time.^44,45^

### The impact of participants’ confidence in informational sources on the level of stress regarding vaccination

One other interesting finding revealed from the analysis of the current survey was the confidence in the informational sources regarding COVID-19 vaccination. As it turns out, participants with a high level of trust in social networks were more susceptible to present vaccination stress. Nevertheless, students with a high level of confidence in scientific journals were significantly less exposed to stress. As a matter of fact, mass media communication has a major role in shaping attitudes towards vaccines.^27^ Prior investigations have stated the existence of an important nexus between social networks, vaccine resistance, and the related conspiracy assumptions.^46,47^ Loomba et al.^48^ quantified the effect of misinformation on vaccination coverage, wherein, compared to real facts, misinformation drove down intent to immunise. This research group demonstrated that science-based misinformation is powerfully associated with a low rate of SARS-CoV-2 vaccine adoption.^48^ In this way, public health organisations could communicate data on vaccination via the official media, referring to scientific studies, thereby increasing the perception of vaccines and reducing their psychological impact on the population.^27^ Besides, healthcare professionals may also contribute to this process, so-called decision support. For example, in a study of pregnant women in New Zealand, decision support was effective in reducing anxiety about immunising their children and raising their intention to undergo vaccination.^11^

### Influencing factors related to COVID-19 vaccination stress

The final logistic regression model suggested that age, institution, housing and confidence level in informational sources concerning SARS-CoV-2 vaccines were the only variables that were significantly associated with stress towards vaccination. We suggest that this could be because of possible interactions amongst explanatory variables, which might eclipse the real relationship between stress as an explained value and predictor variables.

### Limitations of the study

Note that the present work presents some gaps. Given that a cross-sectional study was adopted, the link between stress, willingness to vaccinate, and real future vaccination remains unclear. Another limit concerns the selection of target variables. In the current study, we only examined factors influencing stress over vaccination. It would be possible to reach a more comprehensive understanding of vaccination behaviour by assessing other disorders that may influence it.

## Conclusions

The results presented in our study reflect a positive perception and acceptance of SARS-CoV-2 vaccine, with a considerable level of stress regarding vaccination among Mohammed First University’s students. Indeed, to boost public acceptance of the new vaccines, the country needs to adopt an awareness policy, and transmit clear and accurate scientific information, using the various media that target the entire population (TV, radio, social media, etc.). In addition, healthcare professionals need to be targeted as a key source of information, to convince people about the importance of vaccination.

In the absence of a study addressing potential stress factors associated with immunisation among young Moroccan adults, we assume that these data would be valuable. Our study recommends the improvement of Moroccan young adults’ mental health in terms of better supporting, sensitising and informing them about vaccination at the next booster or other vaccinations.
